# A Neuroimmune Model of Gulf War Illness

**DOI:** 10.15436/2378-6841.17.1665

**Published:** 2017-11-21

**Authors:** Steven S. Coughlin

**Affiliations:** 1 Department of Clinical and Digital Health Sciences, Augusta University, Augusta, GA; 2 Research Service, Charlie Norwood VA Medical Center, Augusta, GA

**Keywords:** Brain imaging, Cytokines, Gulf War syndrome, Gulf War veterans, Human leukocyte antigen, Humoral immunity, Neurocognitive, Serum biomarkers, Symptoms

## Abstract

Several studies have implicated immune system disruption in the pathophysiology of GWI. In addition, alterations in brain structure and functioning have been associated with specific exposures in theater, including pyridostigmine bromide and nerve gas agents. Recent studies conducted up to 25 years after the 1991 conflict have examined factors associated with the continuation or worsening of GWI.

Drawing upon published studies of neural and immune system abnormalities in veterans with GWI, this paper proposes a model of GWI that takes into account neurologic and immunologic pathways, neuroimmune mechanisms of disease pathophysiology, individual predisposition due to sex and genetic background, and comorbid factors including neurological conditions such as neuritis/neuralgia and epilepsy that may occur along a continuum with GWI.

The proposed neuroimmune model of GWI is likely to be useful for designing new research studies, clarifying factors involved in the continuation or worsening of GWI, and identifying biomarker screening algorithms for the illness. The proposed model goes beyond previously proposed frameworks for GWI by taking into account potential differences in risk based upon female *vs.* male sex, time elapsed since exposure to neurotoxicants, duration and severity of illness, comorbid conditions, and genotype.

## Introduction

An estimated 25% to 32% of the 700,000 military personnel who served in the 1991 Gulf War (GW) suffer from Gulf War illness (GWI) (White et al. 2016). GWI illness is sometimes referred to as medically unexplained multisymptom illness or chronic multisymptom illness. Although the cause of GWI is unknown, the most likely etiology is exposure to multiple neurotoxicants such as organophosphate pesticides, sarin or cyclosarin nerve agents, and pyridostigmine bromide medication used as a prophylactic agent against chemical warfare attacks. Although the duration of the conflict was relatively short, the 1991 GW was unusual because of the numbers of chemical exposures experienced by military personnel, including extensive use of pesticides, use of pyridostigmine bromide medication, and low-dose chemical warfare agents released by the destruction of Iraqi facilities (White et al. 2016). The 1991 GW is the only conflict in which pyridostigmine bromide was widely used to protect against the effects of possible nerve gas attacks (Golomb, 1999; White et al. 2016). Military personnel who were deployed to the Persian Gulf commonly experienced multiple exposures in different combinations; those who had some exposures were also much more likely to have additional toxic exposures (Cherry et al. 2001; Boyd et al. 2003; Steele et al. 2012; White et al., 2016). The potential interactive effects of these exposures have not been well-characterized in epidemiologic studies.

Drawing upon published studies of neural and immune system abnormalities in veterans with GWI, including recently reported findings, this paper proposes a model of GWI that takes into account neurologic and immunologic pathways, neuroimmune mechanisms of disease pathophysiology, individual predisposition due to genetic background, and other factors. The proposed neuroimmune model of GWI goes beyond previously proposed frameworks for GWI by taking into account potential differences in risk based upon female *vs.* male sex, time elapsed since exposure to neurotoxicants; duration and severity of illness, and comorbid factors. The latter include neurological conditions such as neuritis/neuralgia and epilepsy that may occur along a continuum with GWI, and chronic, comorbid conditions such as diabetes and cardiovascular disease that can cause fatigue and other symptoms and which become more prevalent as people advance in age.

## Studies of humoral immunity

Several studies have implicated immune system disruption in the pathophysiology of GWI (O’Bryan et al. 2003; Skowera et al. 2004; Whistler et al. 2009; Broderick et al. 2011, 2012, 2013; Smylie et al. 2013; Moss 2013; Parkitney et al. 2015; James et al. 2016; Georgopoulos et al. 2016, 2017; Abou-Donia et al. 2017). Some studies of the role of humoral immunity in GWI have examined the frequency of human leukocyte antigen (HLA) alleles (O’Bryan et al. 2003; Georgopoulos et al. 2016). HLA Class II proteins are located in the Major Histocompatibility Complex MHC of chromosome 6 and are expressed on all nucleated cells. They play a role in immune recognition by presenting peptides from exogenous proteins to CD4+ helper T cells (Meuer et al. 1982). Georgopoulos et al. (2016) identified 144 unique alleles of Class I and II HA genes in 66 veterans with GWI (64 men, 2 women) and 16 without GWI (15 men, 1 woman). These investigators identified 6 Class II alleles that classified participants 84% correctly (56/66 GWI and 13/16 controls). The number of copies of the 6 alleles was significantly higher in the control group, suggesting that the HLA alleles conferred a protective effect for GWI. In support of this interpretation of the findings, overall symptom severity was inversely associated with the number of allele copies. On average, veterans who had larger numbers of allele copies had lower severity of neurological-cognitive-mood, pain, and fatigue symptoms (Georgopoulos et al. 2016). These results are consistent with the hypothesis that exposures of GW veterans served as environmental triggers that contributed to the development of GWI in genetically susceptible veterans who had lower frequencies of HLA alleles that conferred protection. This hypothesized mechanism may relate to both autoimmunity and inflammatory processes since HLA has a role in both (Trowsdale and Knight 2013). The very small number of female research participants precluded looking for sex differences.

James et al. (2016) extended this research by examining whether the effect of HLA allele frequency on GWI symptom severity is exerted by modulating the strength of neural syncronicity. The latter is an important aspect of brain function in health and disease (Singer 1999). Sixty-five GW veterans (63 men, 2 women) with GWI and 16 healthy controls (15 men, 1 woman) underwent a magneto encephalography scan to assess the strength of brain synchronicity. They had previously undergone HLA genotyping to determine the number of copies of 6 protective alleles. The results demonstrated the presence of substantial, widespread HLA- and non-HLA-related neuronal influences on neurological-cognitive-mood, pain, and fatigue symptom severity in GWI (James et al. 2016). The study was limited by the small number of female research participants.

Georgopoulos et al. (2016) compared synchronous neural interactions between patients with GWI (n = 40) and seven other diseases (schizophrenia [n = 21], Alzheimer’s disease [n = 66], posttraumatic stress disorder [n = 159], major depressive disorder [n = 10], relapsing-remitting multiple sclerosis [n = 43], Sjogren’s syndrome [n = 32], and rheumatoid arthritis [n = 8]). All of the research participants underwent a resting-state magneto encephalographic scan to calculate synchronous neural interactions. Synchronous neural interactions observed in patients with GWI differed significantly from those observed in patients with schizophrenia, Alzheimer’s disease, posttraumatic stress disorder, and major depressive disorder but not from those in patients with relapsing-remitting multiple sclerosis, Sjogren’s syndrome, and rheumatoid arthritis. Because GWI brain synchronicity did not differ significantly from that of the three immune-related diseases (relapsing-remitting multiple sclerosis, Sjogren’s syndrome, and rheumatoid arthritis) but did differ significantly from that of the other diseases, these findings indicate that altered brain communication in GWI likely reflects immune-related processes (Georgopoulos et al. 2017; James et al. 2016). The number of female research participants was not reported.

## Autoantibodies to neuronal and glial proteins

The cytoarchitecture of the central nervous system (CNS) is maintained by a complex cellular milieu that includes neuronal and glial cells that maintain proper communication if functioning properly. Abou-Donia et al. (2017) screened the serum of 20 veterans with GWI (8 males, females) and 10 non-veteran controls (4 males, 6 females) who had low-back pain for the presence of auto antibodies to neuronal and glial proteins (neurofilament triplet proteins, tubulin, microtubule associated tau proteins, microtubule associated protein-2, myelin basic protein, myelin associated glycoprotein, glial fibrillary acidic protein, calcium-calmodulin kinse II, and glial S-100B protein). Tau proteins are involved in the stabilization and assembly of axonal microtubules (Liliang et al. 2011). Myelin basic protein is produced by oligodendroglia in the (CNS) and Schwann cells in the peripheral nervous system (Abou-Donia et al. 2017). Glial fibrillary acidic protein plays an important role in maintaining the shape and motility of astrocytic processes and contributes to white matter architecture, myelination, and blood brain barrier integrity (O’Callaghan et al. 2015). In the study by Abou-Eonia et al. (2017), veterans with GWI had significantly higher levels of autoantibody reactivity in all proteins examined except S-100B. If these findings are replicated in larger samples of veterans, serum autoantibodies may be useful as biomarkers of GWI. The presence of neuronal injury/gliosis in GW veterans is consistent with results from epidemiologic studies indicating that 25 years after the 1991 conflict, the health of many veterans with GWI is not improving and may be getting worse (Abou-Donia et al. 2017; White et al. 2016).

## Immune network signaling

Whistler et al. (2009) conducted a pilot study involving 9 GWI cases and 11 sedentary control veterans who had not been deployed to the Persian Gulf. The cases and controls were matched on age and body mass index. Natural killer cell (NK) cytotoxicity, cytokines (tissue necrosis factor α, interleukin (IL)-10, IL-6, IL-5, IL-α, interferon Y), and expression levels of 20,000 genes were measured immediately before, immediately after, and 4 hours following a standard bicycle ergometer exercise challenge. Significant differences were observed in three NK cell subsets and NK cytotoxicity. Veterans with GWI had impaired immune function as shown by decreased NK cytotoxicity and altered gene expression associated with NK cell function. In addition, pro-inflammatory cytokines and dysregulated mediators of the stress response, with low baseline salivary cortisol, were altered in GWI cases compared to controls. The exercise challenge augmented these differences. A further finding was that genes distinguishing cases from controls were associated with the glucocorticoid signaling pathway. The findings of Whistler et al. (2009) suggest a role for immune cell dysfunction in sustaining GWI over time.

In a pilot study involving 9 GWI cases and 11 control subjects (numbers of male and female subjects not reported), Broderick et al. (2011) examined whether GWI presents with a distinct pattern of immune signaling. Rather than examining cytokines in isolation, these investigators examined the patterns of interaction of cytokines and other immune markers in an attempt to identify a characteristic GWI signature. Blood was collected prior to a graded exercise test, at peak effort and 4 hours post-exercise. Salivary cortisol and plasma, serum or culture supernatants were analyzed for concentration of neuropeptide Y, tissue necrosis factor α, interleukin (IL)-10, IL-6, IL-5, IL-1α, interferon Y, and soluble CD26. Mutual information networks linking these immune markers were generated in each group at each time point. Graph theory was then used to describe each network’s structure. GWI networks had more connections but were less organized. Under effort, significant restructuring was observed around nodes for CD19+ β cell population, IL-5, IL-6, and soluble CD26 concentrations. In addition, IL-1α and CD2+CD26+ nodes strongly influencedB and T cell network patterns. These results indicating altered cytokine signaling are consistent with an autoimmune component in the etiology of GWI. Immune processes involving cytotoxic T cells and NK cells may be temporarily disrupted by normal responses to exercise such as the release of cortisol. IL-5 promotes B cell growth and immunoglobulin secretion. CD26 plays a key role in T cell-dependent antibody production and the switching of immunoglobulin isotype in B cells and also contributes to the regulation of CD4+ T cells and NK cells (Broderick et al. 2011).

Broderick et al. (2011) extended their research on patterns of immune signaling in GWI in a study of 20 male GWI cases, 22 healthy veterans, and 7 patients with chronic fatigue syndrome (CFS). Like in the earlier study (Broderick et al. 2009), blood was drawn during a graded exercise test, prior to exercise, at peak effort, and 4-hours post exercise. Circulating lymphocytes collected at each time point were analyzed for gene expression using a conventional single-gene approach and also with a novel approach whereby transcript levels were projected onto the regulatory circuitry of known pathways. Significant increases in immune-neuroendocrine signaling and inflammatory activity and down-regulation of apoptotic signaling were observed in GWI cases.

In addition to clarifying the pathophysiology of GWI and factors that contribute to the continuation or progression of the illness over time, studies of biomarkers of immune function may also help to identify biomarkers of GWI. Broderick et al. (2012) examined the coexpression of 12 biomarkers of immune and endocrine function in a study of 26 GWI patients, 13 health controls, and 9 unhealthy controls with CFS. All of the subjects were male. Blood was collected at 3 time points during a graded exercise challenge. The 3 groups were separated almost completely on the basis of two coexpression patterns (sensitivity 70%, specificity 90%).

Smylie et al. (2013) compared sex-specific immune signatures in GWI and CFS. Blood was drawn during a graded exercise test, prior to exercise, at peak effort, and 4-hours post exercise. The concentrations of tissue necrosis factor β, tissue necrosis factor α, IL-23, 17, 15, 13, 12, 10, 6, 5, 4, 2, 1α, and interferon Y were measured at each time point. Using between 2 and 5 cytokine markers, classification accuracies in excess of 80% were observed. In male subjects, IL-10 and IL-23 expression contributed in an illness and time-dependent manner in the context of IL-15, 12, 2, and interferon Y. In contrast, IL-10 was identified as a delineator in the context of IL-17, 5, and 4 in female subjects with GWI or CFS. These findings are consistent with the involvement of the IL-23/IL-17 axis in a sex-specific manner. Overlap between IL-23 as a marker of GWI and its contribution as a marker of sex-specific response to exercise suggests the involvement of the sex hormone axis in modulating the IL-23/IL-17 immune response (Smylie et al. 2013).

## Brain Imaging Studies

Alterations in brain structure and functioning have been associated with specific exposures in theater, including pyridostigmine bromide and nerve gas agents. Neuroimaging studies employing magnetic resonance imaging (MRI), functional MRI (fMRI) and other sophisticated noninvasive imaging techniques have demonstrated abnormalities in brain tissues in veterans with GWI (Haley et al. 2009; Calley et al. 2010; Li et al. 2011; Chao et al. 2011; Rayhan et al. 2013; for a recent review, see White et al. 2016). As an example, in the fMRI study by Rayhan et al. (2013), GW veterans with GWI or CFS were found to have white matter integrity loss in cortio-cortical and cortiospinal areas. In the MRI study by Chao et al. (2011), GW veterans exposed to sarin and cyclosarin had significantly reduced total gray and white matter volumes compared to unexposed controls.

## Neurocognitive Abnormalities

Neurocognitive research involving GW veterans has consistently shown exposure-related dysfunction among GW veterans who were deployed to the Persian Gulf. The abnormalities identified in these studies (for example, poorer visuospatial and motor skills) among GW veterans who were exposed to neurotoxicants such as sarin and pyridostigmine bromide during deployment) are consistent with structural and functional pathology of the central nervous system (Axelrod et al. 1997; Anger et al. 1999; Binder et al. 1999; Storzbach et al. 2001; Sullivan et al. 2003; Proctor et al. 2006). GWI symptoms commonly include complaints about memory and concentration as well as dysregulated mood (White et al. 2016).

## Proposed Neuroimmune Model of Gulf War Illness

**Figure 1:**
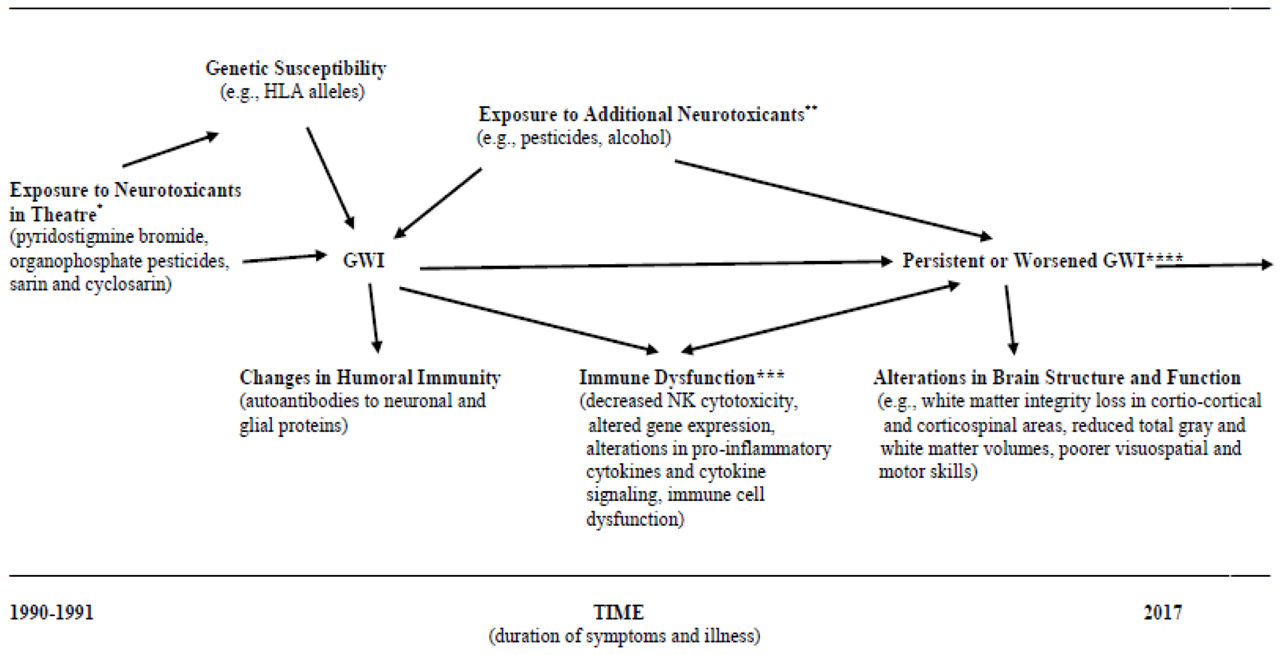
Proposed Neuroimmune Model of Gulf War Illness. *Initial exposures at the time of the 1991 conflict that may have precipitated GWI. **More recent exposures that may worsen symptoms and illness. ***Sex hormones may modulate the proinflammatory cytokine immune response. Immune dysfunction may contribute to persistent GWI. ****Persistent GWI can co-occur with neurological conditions such as neuritis/neuralgia and epilepsy that may occur along a continuum with GWI, and with comorbid conditions such as diabetes and cardiovascular disease that can cause fatigue and other symptoms and which become more prevalent with age. comorbid factors including neurological conditions such as neuritis/neuralgia and epilepsy that may occur along a continuum with GWI, and chronic, comorbid conditions such as diabetes and cardiovascular disease that can cause fatigue and other symptoms and which become more prevalent as people advance in age.

The proposed neuroimmune model of GWI is shown in Figure 1. This model is based upon a large body of research involving GW veterans suffering from GWI and is also supported by animal studies (for a recent review of animal studies of GWI see White et al. 2016). Likely exposures to neurotoxicants in theatre are shown on the left-hand side of the figure. The model allows for the possibility that veterans suffering from GWI may have been exposed to multiple known or unknown neurotoxicants including organophosphate pesticides, sarin or cyclosarin nerve agents, and pyridostigmine bromide. Genetic susceptibility is shown on the upper left-hand side. This includes genetic factors that may modulate the effects of environmental exposures such as number of copies of specific HLA alleles (O’Bryan et al. 2003; Georgopoulos et al. 2016). As another example, Steele et al. (2015) observed a gene-exposure interaction involving butyryl cholinesterase genotype and enzyme activity in a case-control study of Gulf War veterans. The proposed model is therefore relevant to studies that test the hypothesis that exposures of GW veterans at the time of the 1991 conflict served as environmental triggers that contributed to the development of GWI in genetically susceptible veterans who had lower frequencies of HLA alleles that conferred protection.

Changes in humoral immunity such as autoantibodies to neuronal and glial proteins are highlighted in the model as they may serve as useful biomarkers of GWI (Abou-Donia et al. 2017). Currently, there are no universally accepted and validated biomarkers for GWI. This is an area of active research which could contribute importantly to the diagnosis of GWI and to the selection of therapeutic interventions.

Additional aspects of immune dysfunction are shown in the middle of the figure, towards the bottom (Figure 1). This includes decreased NK cytotoxicity, altered gene expression, alterations in pro-inflammatory cytokines, altered cytokine signaling, and immune cell dysfunction (Whistler et al. 2009; Broderick et al. 2011 Broderick et al. 2012; Smylie et al. 2012). The arrow indicating the linkages between immune dysfunction and persisten GWI is bidirectional and immune cell dysfunction may have an important role in sustaining GWI over time (Whistler et al. 2009). The model takes into account the fact that sex hormones may modulate the proinflammatory cytokine immune response (Smylie et al. (2013).

Exposures to neurotoxicants that may have occurred long after the 1991 conflict are shown in the middle of the figure, at the top. Men and women are potentially exposed to a number of neurotoxicants in daily life including pesticides used in gardening, consumption of alcohol, and solvents used in some occupations. Little is known about the potential adverse effects of such environmental exposures among GW veterans who suffer from GWI. It is conceivable that exposure to such neurotoxicants could worsen GWI or contribute to its persistence over long periods of time. Thus, the model highlights the need for additional research in this area, which could contribute to the development of evidence-based guidelines for veterans suffering from GWI and their health care providers who counsel them.

Finally, the model highlights alterations in brain structure and function such as white matter integrity loss in corti-co-cortical and corticospinal areas, reduced total gray and white matter volumes (in GW veterans exposed to sarin and cyclosarin), and poorer visuospatial and motor skills (Figure 1). Such abnormalities have been observed in neuroimaging studies employing MRI, functional MRI, fMRI, and other sophisticated noninvasive imaging techniques (Haley et al. 2009; Calley et al. 2010; Li et al. 2011; Chao et al. 2011; Rayhan et al. 2013). Neurocognitive studies have shown exposure-related abnormalities such as poorer visuospatial and motor skills which are consistent with structural and functional pathology of the central nervous system (Axelrod et al. 1997; Anger et al. 1999; Binder et al. 1999; Storzbach et al. 2001; Sullivan et al. 2003; Proctor et al. 2006).

## Conclusions

The proposed neuroimmune model of GWI is likely to be useful for designing and planning new research studies of GWI, clarifying factors involved in the continuation or worsening of GWI, and identifying biomarker screening algorithms for the illness. The proposed model goes beyond previously proposed frameworks for GWI by taking into account potential differences in risk based upon female vs. male sex, time elapsed since exposure to neurotoxicants, duration and severity of illness, genotype, and comorbid conditions including neurological conditions such as neuritis/neuralgia and epilepsy that may be on a continuum with GWI. As GW veterans advance in age, they are increasingly at risk of chronic conditions such as diabetes and cardiovascular disease that can cause fatigue and other symptoms and which can co-occur with GWI (Coughlin et al. 2013). There is a pressing need for research to identify validated biomarkers of GWI in order to identify veterans suffering from both GWI and comorbid conditions and to facilitate the identification of effective therapeutic interventions. The proposed model highlights these important issues.

Results from longitudinal studies suggest that rates of GWI and symptoms reported by GW veterans are fairly constant with little or no improvement over time (Wolfe et al. 2002; Hotopf et al. 2003; Ozakinci et al. 2006). The proposed model takes into account time elapsed since exposure to neurotoxicants, the potential for additional exposures to neurotoxicants long after the 1991 conflict, and the duration and severity of GWI. It is possible that alterations in brain structure may be more likely to occur among veterans who have suffered from GWI over long periods of time or who have more severe illness.

Finally, the proposed model highlights the fact that sex-differences in GWI may occur and that sex hormones may modulate the proinflammatory cytokine immune response (Smylie et al. 2013). Unfortunately, many of the clinical research studies reviewed in this article were limited to male veterans or had too few women veterans for separate analysis. The study by Smyulie et al. (2013) is an important exception. Additional studies are needed that explore potential sex-differences in risk of GWI (Coughlin 2016; Coughlin et al. 2017).
